# Effect of High Hydrostatic Pressure Processing on the Anthocyanins Content, Antioxidant Activity, Sensorial Acceptance and Stability of Jussara (*Euterpe edulis*) Juice

**DOI:** 10.3390/foods10102246

**Published:** 2021-09-22

**Authors:** Andressa Alves de Oliveira, Alexandre Guedes Torres, Daniel Perrone, Mariana Monteiro

**Affiliations:** 1 Laboratório de Alimentos Funcionais, Nutrition Institute, Federal University of Rio de Janeiro, Av. Carlos Chagas Filho, 373, CCS, Bloco J, 2° Andar, Sala 16, Rio de Janeiro 21941-902, Brazil; oliveira.anutri@gmail.com; 2 Laboratório de Bioquímica Nutricional e de Alimentos, Biochemistry Department, Chemistry Institute, Federal University of Rio de Janeiro, Av. Athos da Silveira Ramos 149, CT, Bloco A, Sala 528A, Rio de Janeiro 21941-909, Brazil; torres@iq.ufrj.br

**Keywords:** antioxidants, HHP, phenolic compounds

## Abstract

Jussara (*Euterpe edulis*) fruit is a strong candidate for exportation due to its high content of anthocyanins. However, its rapid perishability impairs its potential for further economic exploration, highlighting the relevance of producing ready-to-drink juices by applying innovative processing, such as high hydrostatic pressure (HHP). The effect of HHP (200, 350, and 500 MPa for 5, 7.5, and 10 min) on anthocyanins content and antioxidant activity (AA) by FRAP and TEAC assays, and the most effective HHP condition on overall sensory acceptance and stability of jussara juice, were investigated. While mild pressurization (200 MPa for 5 min) retained anthocyanins and AA, 82% of anthocyanins content and 46% of TEAC values were lost at the most extreme pressurization condition (500 MPa for 10 min). The addition of 12.5% sucrose was the ideal for jussara juice consumer acceptance. No significant difference was observed for overall sensory acceptance scores of unprocessed (6.7) and HHP-processed juices (6.8), both juices being well-accepted. However, pressurization was ineffective in promoting the retention of anthocyanins and AA in jussara juice stored at refrigeration temperature for 60 days, probably due to enzymatic browning.

## 1. Introduction

In the last few years, the Brazilian fruit juice industry has undergone an expansion, especially due to the expansion of international markets for tropical fruit juices exhibiting high nutritional value as well as exotic flavors. The jussara palm (*Euterpe edulis*), native to the Atlantic rainforest and similar to the açaí palm (*Euterpe oleracea*), but not as commercialized and consumed, produces a round fruit that has a single seed covered by a thin, shiny, purple- to black-colored layer, which yields a pulp often used to prepare varied beverages and ice creams. Previous work from our research group [1,2] and others [3,4,5] reported that jussara pulp shows both relevant nutritional value—a dietary source of fiber, copper, manganese, tocopherols, β-carotene, and unsaturated fatty acids—and functional potential, including high contents of anthocyanins, mainly cyanidin-3-*O*-glucoside and cyanidin-3-*O*-rutinoside.

Despite its great nutritional and functional potential, the rapid perishability of the fruit hampers its high-scale commercialization. Jussara depulping industries usually commercialize the frozen pulp, which is not generally directly consumed, requiring additional steps such as thawing, addition of sugars and other ingredients (e.g., guaraná syrup or fruits such as bananas, strawberries, guava, etc.), and mixing. To produce ready-to-drink juices, an innovative processing method is high hydrostatic pressure (HHP), a conservation method that enables chilled storage of processed juices. HHP involves subjecting foods to high isostatic pressure (from 100 to 1000 MPa), causing a number of changes in the morphology, cell membrane, or biochemical reactions of microorganisms. All these effects are related to the inactivation of microorganisms, and compared to classical thermal conservation methods such as pasteurization, HHP process tend to preserve the nutritional, functional, and sensorial qualities of perishable foods [6,7]. However, a limitation of HHP is that resistant bacterial spores and browning enzymes require a very high pressure to become inactive, more than 1000 MPa, or the associated pressure given a certain temperature [6]. The application of HHP to fruit products has been considered the most effective and realistic because the inherently low pH of fruits can inhibit the growth of most spoilage bacteria. Furthermore, the yeast and mold that survive such low pH ranges are relatively susceptible to HHP [6]. Another advantage of this process in fruit products is that fruits are rich in phenolic compounds, and many of these compounds are lost in heat treatment [8]. 

The effects of HHP processing on phenolic compounds are complex. Many factors, such as phenolic compounds’ individual pressure sensitivity, their interaction with other matrix constituents, HHP parameters, and packing materials could influence the contents of phenolic compounds in pressurized food products [9]. Some works reported that HHP caused decreases of up to 25% in anthocyanins content in pomegranate juice [10] and strawberry purée [11]. It has been shown that HHP processing preserves anthocyanins in strawberry pulp (400 MPa for 5 min) [12] and juice (400 MPa for 3 min) [13], and even increases their contents by up to 37% in blue honeysuckle pulp (200 MPa for 5 min) [14], blueberry juice (200 MPa for 5 min) [15], and jabuticaba juice (200 MPa for 5 min) [16].

Since the effects of HHP processing on food products depend on their matrix, this is the first study aiming at investigating the effect of high hydrostatic pressure processing on anthocyanins content, antioxidant activity, and consumer acceptance of jussara juice. In addition, anthocyanins and antioxidant activity retention were determined during refrigerated storage for 60 days. 

## 2. Materials and Methods

### 2.1. Samples

Commercial jussara juice (Euterpe edulis) was produced by Juçaí^®^ processing company, Resende, Brazil. Fruits were selected, washed, and sanitized in 100 ppm sodium hypochlorite solution for 15 min. Then, jussara fruits were submerged in water at approximately 40 °C for 30 min and subsequently depulped in a vertical depulper with additional water (1 L of water per 2.5 kg of fruit). Jussara juice presented approximately 3 °Brix and a pH of 4.8. The juice was packed in aseptic polyethylene bags and stored at −20 °C.

### 2.2. High Hydrostatic Pressure Processing

Fifty milliliters of frozen jussara juice (−20 °C) was thawed, transferred to a small five-layer nylon bag with an oxygen barrier, and vacuum-sealed (TecMaq^®^, São Paulo, Brazil). These bags were submitted to HHP processing in a pilot equipment (Bras Solution Ltd., Rio de Janeiro, Brazil). The high pressure vessel (1000 × 700 × 600 mm, height × width × length, respectively) was filled with distilled water and a concentrated carboxylate-based synthetic fluid (2:1, *v*/*v*) as a pressure-transmitting medium. Both this fluid and the juice samples were at room temperature (27 °C). Pressure (200, 350, and 500 MPa) and time of processing (5, 7.5, and 10 min) were defined according to a 2^2^ full factorial design with a central point, in duplicate, totaling 10 experimental runs (Table S1). After pressurization, juice samples were frozen until analysis. The response variables in this experimental design were anthocyanins content determined by HPLC with diode array detection (HPLC-DAD) and antioxidant activity (AA) assessed by Ferric reducing antioxidant power (FRAP) and Trolox equivalent antioxidant capacity (TEAC) assays.

### 2.3. Anthocyanins Analysis

Jussara juice was analyzed directly after centrifugation (11,300× *g*, 10 min) and filtration through a 0.45 µm PTFE filter unit (Millipore^®^, Barueri, Brazil). Samples were diluted (1:1, *v*/*v*) with 1% aqueous formic acid. The liquid chromatography system (Shimadzu^®^, Kyoto, Japan) included a quaternary pump LC-20AT, automatic injector SIL-20AHT, diode array detector (DAD) SPD-M20A, system controller CBM-20A, and degasser DGU-20A5. Chromatographic separation of cyanidin-3-O-glucoside (C3G) and cyanidin-3-O-rutinoside (C3R) was achieved using a reversed-phase column C18 (5 μm, 250 mm × 4.6 mm, Kromasil^®^), according to Inada et al. [16]. Chromatographic peaks’ identities were assigned by comparison with the retention times and absorption spectra of the respective standards. Quantification was performed by external calibration. Data were acquired by LC solution software (Shimadzu Corporation^®^ Kyoto, Japan, version 1.25, 2009).

### 2.4. Antioxidant Activity

The AA of jussara juice was assessed by FRAP and TEAC assays directly after centrifugation (11,300× *g*, 10 min) of jussara pulp, using the supernatants. Each sample was analyzed in triplicate. The FRAP assay was performed according to Benzie and Strain [17] with slight modifications, as described by Inada et al. [16]. The results are expressed as micromoles of Fe^2+^ equivalents per 100 mL of juice. The TEAC assay was performed according to Re et al. [18], with slight modifications, as described by Inada et al. [16]. The results are expressed as micromoles of Trolox equivalents per 100 mL of juice.

### 2.5. Sensorial Analysis

An ideal sucrose concentration test of jussara juice was carried out with a group of 99 untrained consumers (55 women and 44 men) aged between 18 and 69 years, on average 30 years, habitually consuming fruit juices at least twice a week, recruited among staff, students, and visitors at the Federal University of Rio de Janeiro, Brazil. Only consumers who reported frequent consumption of fruit juices (at least twice a week) were selected to participate in this test. Unprocessed jussara juice was sweetened with six different sucrose concentrations: 2.5%, 5.0%, 7.5%, 10.0%, 12.5%, and 15.0%. Each consumer evaluated sweetness using the just-about-right (JAR) scale, with intensity ranging in centimeters (cm) from 0 (“extremely less sweet than ideal”) to 9 (“extremely more sweet than ideal”), with “ideal sweetness” specified as 4.5 cm. Jussara juice samples were offered to consumers at refrigeration temperature in 50 mL plastic cups coded with three-digit numbers. The samples were presented in a monadic sequential random order and each consumer evaluated all six jussara juice samples of different sucrose concentrations. The results were analyzed by linear regression analysis of the score given by the consumers for each different sucrose concentration.

After sucrose addition at 12.5%, previously determined as the ideal sucrose concentration, half of the juice volume was pressurized at 200 MPa for 5 min. Unprocessed and HHP-processed jussara juice were stored at 4 °C for two days prior to sensory analysis. An overall impression test was carried out with a group of 80 untrained consumers (47 women and 33 men) aged between 17 and 63 years, on average 29 years, recruited among staff, students, and visitors at the Federal University of Rio de Janeiro, Brazil. Only consumers who reported frequent consumption of fruit juice (at least twice a week) and who enjoyed açaí juice were selected to participate in this test. Each consumer evaluated the overall impression using an unstructured (9 cm) scale, with intensity ranging from 0 (“dislike extremely”) to 9 (“like extremely”), with “indifferent” specified as 4.5 cm. Jussara juice samples were offered to consumers at refrigeration temperature in 50 mL plastic cups coded with three-digit numbers. Unprocessed and HHP-processed jussara juices were presented in a monadic sequential random order.

### 2.6. Juices Stability

The anthocyanins content and AA (FRAP and TEAC) of unprocessed and HHP-processed (200 MPa for 5 min) juices (both unsweetened) were evaluated during refrigerated storage in the dark, at baseline and after 15, 30, 45, and 60 days of storage. Analyses were performed as previously described.

### 2.7. Statistical Analysis

The experimental design matrix was generated and analyzed using Statistica software, version 7.0 (StatSoft Inc., Tulsa, OK, USA). Analysis of variance (one-way ANOVA) followed by Dunnett’s multiple comparison post hoc test was used for comparing the anthocyanins content and antioxidant activity of unprocessed and HHP-processed juices. As the overall impression scores did not present a normal distribution, comparison of unprocessed and HHP-processed juices was performed using a Wilcoxon matched-pairs signed-ranked test. Repeated measures two-way ANOVA followed by Sidak’s multiple comparisons test were used to investigate anthocyanins and AA retention during storage. Statistical analyses were performed using GraphPad Prism software for Windows, version 5.04 (GraphPad Software, San Diego, CA, USA). Differences were considered significant when *p* < 0.05.

## 3. Results and Discussion

### 3.1. Mild Pressurization Conditions Did Not Affect Anthocyanins and Antioxidant Activity of Jussara Juice

**Figure 1:**
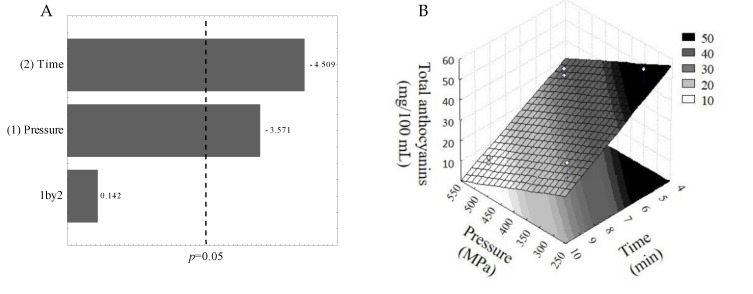
(**A**) Total anthocyanins content in jussara juice was affected by both pressure and time by HPP processing. In this Pareto chart, the standardized effects that reached the dotted line (*p* = 0.05) are statistically significant. (**B**) Surface response chart obtained from the experimental design results, showing the total anthocyanins content as a function of HHP processing conditions.

The major anthocyanins reported for jussara [1,3], C3G and C3R, were identified and quantified in juice samples (Figure S1). We observed that the total anthocyanins content of jussara juice was negatively affected by both HHP processing conditions (pressure and time) (Figure 1A). Therefore, highest anthocyanins content was found at lower pressures and shorter processing times (Figure 1B).

**Figure 2:**
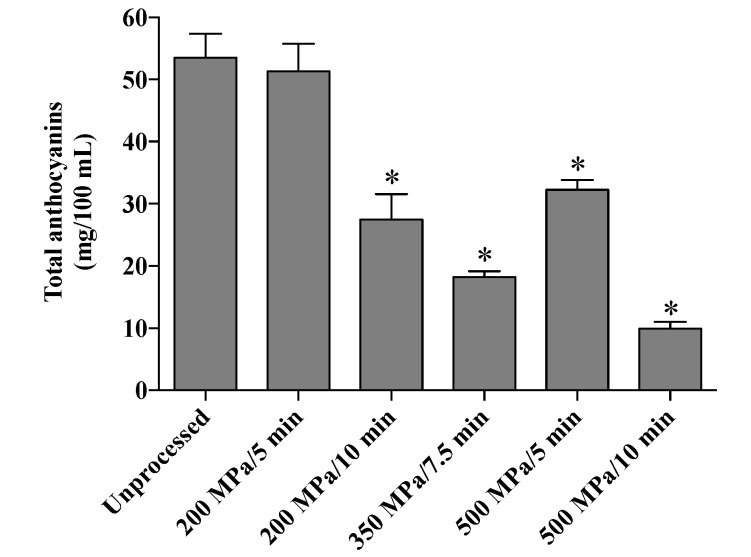
Total anthocyanins content (mg/100 mL) in unprocessed and HHP-processed jussara juices. Asterisks indicate significant differences from the unprocessed juice (one-way ANOVA followed by Dunnett’s post hoc test, *p* < 0.05).

In fact, the only HHP processing condition that did not affect anthocyanins content was 200 MPa for 5 min (Figure 2). Significant (*p* < 0.05) losses of 49%, 66%, 40%, and 82% in total anthocyanin content were observed upon pressurization at 200 MPa/10 min, 350 MPa/7.5 min, 500 MPa/5 min, and 500 MPa/10 min, respectively.

Terefe et al. [11] observed that HHP processing (600 MPa/5 min) of strawberry purées resulted in up to 28% loss of anthocyanins, similar to the result of the 500 MPa/5 min condition in our study. In the literature, the effects of pressurization on anthocyanins content in fruits are conflicting, being affected by several factors. One of these is the food matrix, which could lead to different behaviors when identical processing conditions are employed to different fruits. For instance, Patras et al. [19] reported that pressurization at 400 MPa for 15 min led to a 3% increase in the anthocyanins content of blackberry purée, whereas a 14% decrease was observed for strawberry purée. Moreover, if we compare the results reported by Cao et al. [12] for strawberry (no effect on anthocyanins content) with those reported by Barba et al. [15] for blueberry juice (15% increase), by Inada et al. [16] for jabuticaba juice (7% decrease), and by Liu et al. [14] for blue honeysuckle berry (7% decrease), we can observe that the food matrix is relevant to the HHP processing end result.

Another relevant factor that affects anthocyanins retention in HHP-processed fruits is the processing conditions. When the same fruit is subjected to different pressures and times of processing, the effect on the anthocyanins content may vary, sometimes increasing, other times decreasing, or even remaining unaltered. Liu et al. [14] reported an increase of up to 7% when blue honeysuckle berry was pressurized at 200 MPa for 5 or 10 min. On the other hand, when more intense processing conditions were employed (400 MPa/20 min, 500 MPa/15 min, and 600 MPa/10 min), losses of 7% were observed. When blackberry and strawberry purées were pressurized for 15 min at 400 MPa, changes in anthocyanins content were observed (3% increase and 14% decrease, respectively), whereas at higher pressures (500 and 600 MPa) no change was observed [19]. The same behavior in terms of effect on anthocyanins content was observed for blueberry [15] and jabuticaba juices [16].

**Figure 3:**
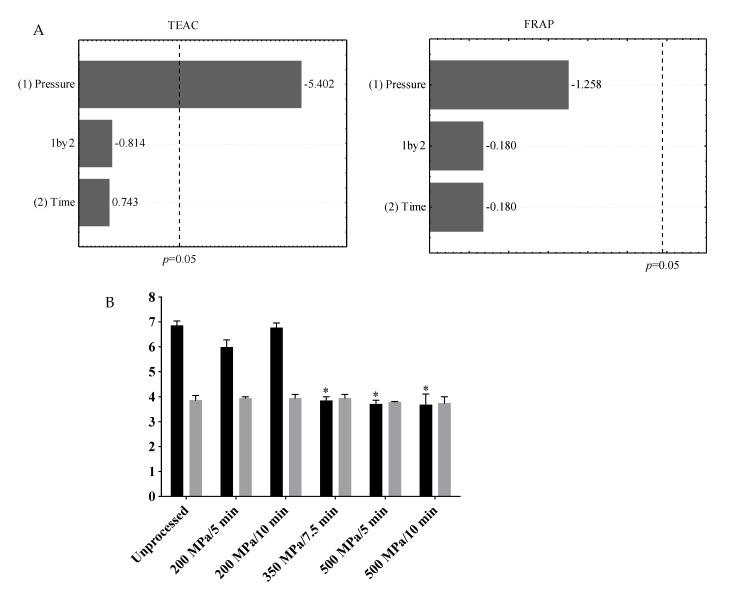
(**A**) Antioxidant activity of jussara juice assessed by TEAC, but not by FRAP, was affected by pressure during HHP processing. In these Pareto charts, the standardized effects that reached the dotted line (*p* = 0.05) are statistically significant. (**B**) Antioxidant activity assessed by TEAC (■; mmol Trolox/100 mL) and FRAP (■; mmol Fe^2+^/100 mL) assays of unprocessed and HHP-processed jussara juices. Asterisks indicate significant differences from the unprocessed juice (one-way ANOVA followed by Dunnett’s post hoc test, *p* < 0.05).

Increases in anthocyanins content are usually associated with a higher extractability caused by pressurization [20], which damages the integrity of plant cell walls’ microstructures [21]. Decreases in anthocyanins content due to HHP processing may be explained by either activation of polyphenol oxidase (PPO, EC 1.10.3.1) and peroxidases (POD, EC 1.11.1.x) or adiabatic heating [14]. Moreover, the comparison of studies in the literature should be made with caution, considering that some studies employed HPLC analysis whereas others used less specific spectrophotometric methods. In summary, the effect of HHP on anthocyanins content in berries cannot be generalized, since food matrix and processing conditions play a relevant role. The only processing variable that influenced AA was pressure, which negatively affected TEAC, but not FRAP values (Figure 3A). In that sense, pressures of 350 MPa or higher led to an average loss of 45.3% of TEAC values, whereas no difference was observed between unprocessed and HHP-processed jussara juices in the FRAP values (Figure 3B).

Similarly, several studies reported reduction in TEAC values after HHP processing of strawberry purées, and orange and blueberry juices [15,19]. As expected, total anthocyanins content was positively correlated with TEAC values (*r* = 0.69, *p* = 0.0135, *n* = 12), whereas no correlation was observed for FRAP values, suggesting that TEAC assay was able to measure the AA of jussara juice related to the presence of anthocyanins. In summary, pressurization of jussara juice at 200 MPa for 5 min preserved both anthocyanins content and AA.

### 3.2. Sucrose Addition and HHP Processing Yielded a Well-Accepted Jussara Juice 

**Figure 4:**
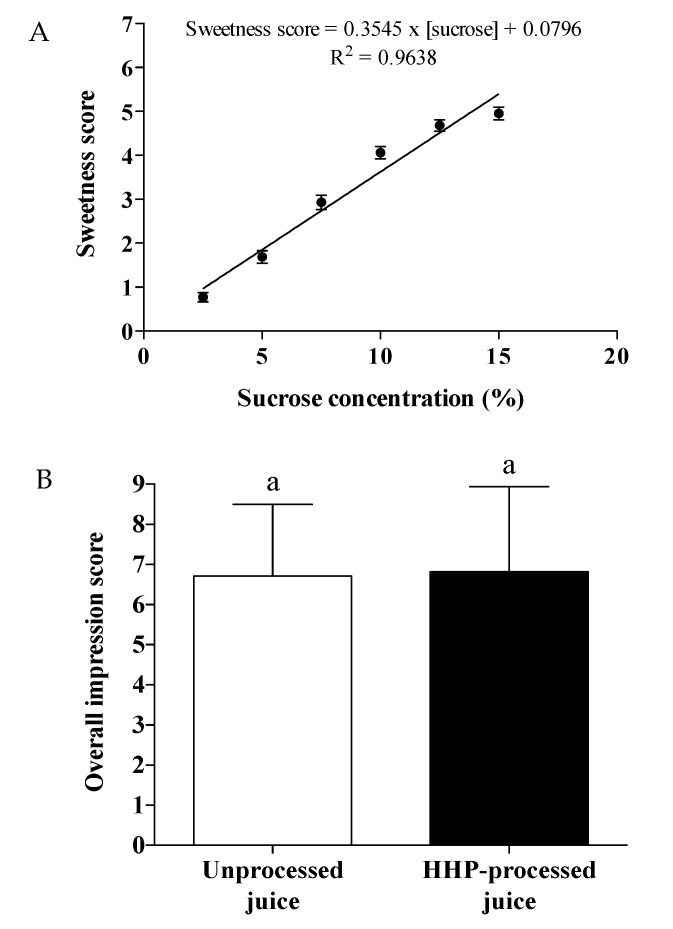
(**A**) The ideal concentration of added sucrose in jussara juice (12.5%) was determined from the linear regression analysis of the sweetness score (mean ± standard error of the mean; *n* = 99). (**B**) No significant difference was observed between the overall impression score of sweetened unprocessed and HHP-processed (200 MPa for 5 min) jussara juice (Wilcoxon matched-pairs signed-rank test, *n* = 80).

The ideal level of sucrose addition to jussara juice was determined in a sensory test with 99 consumers. The proposed linear model showed an excellent fit, explaining more than 96% of the variance (Figure 4A). The ideal sucrose concentration for addition in jussara juice was 12.5%, slightly higher than that reported for passion fruit juice (9.4% to 10.0%) [22], mixed juices from Amazon fruits (9.5% to 10.7%) [23], papaya and pitanga nectars (10.0%) [24,25], and peach nectar (10.4%) [26]. Since jussara juice presents a relatively high lipid content (7.5%) [1] and a distinctive earthy flavor [27], a higher ideal sucrose concentration was expected. These differences reinforce the need of experimentally determining the ideal sucrose concentration when developing a specific food product.

No significant difference was observed between the overall impression scores for unprocessed (6.7 ± 1.8) and HHP-processed (200 MPa/5 min) (6.8 ± 2.1) jussara juices (Figure 4B), indicating that both juices were well-accepted, with mean scores equivalent to “like moderately”. Even though we did not investigate the specific sensory attributes of the juices in the present work, some consumers spontaneously reported that HHP-processed juice had a more homogeneous texture than the unprocessed juice, probably due to the effect that pressurization had on suspended particles. Moreover, since jussara juice aroma is not intense and fragrant, HHP processing probably did not affect this attribute, leading to its sensorial acceptance. It is known that HHP processing at low temperatures prevents the formation of off flavors related to the Maillard reaction, as opposed to thermal processing [28].

### 3.3. HHP Processing on Retention of Anthocyanins Content and Antioxidant Activity of Jussara Juice during Refrigerated Storage for 60 Days 

**Figure 5:**
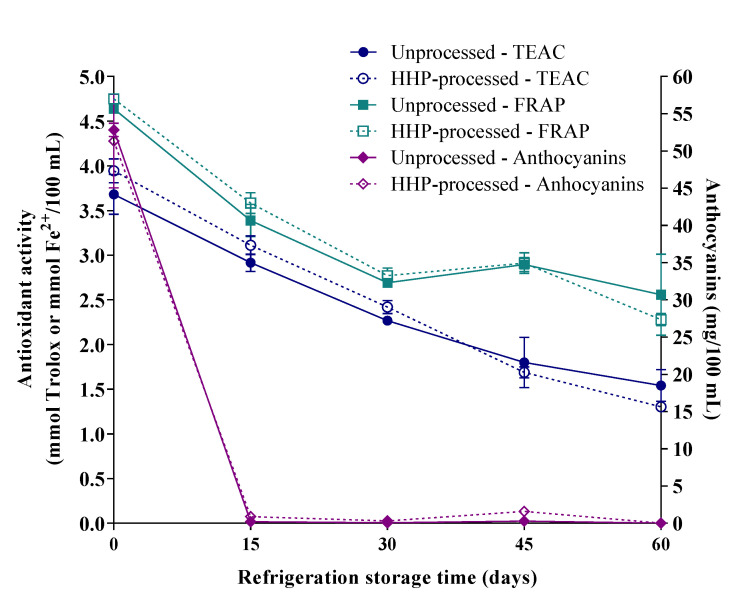
Storage of jussara juice for 60 days of refrigeration caused losses of both anthocyanins and antioxidant activity, assessed by FRAP and TEAC assays (*p* < 0.0001). While HHP processing at 200 MPa for 5 min affected the loss of TEAC values during storage (*p* = 0.021), no effect was observed for the losses of anthocyanins and FRAP values (*p* > 0.780) (repeated measures two-way ANOVA).

Anthocyanins content in jussara juices decreased drastically (98%, on average) in the first 15 days during refrigerated storage (Figure 5, *p* < 0.0001), indicating that HHP processing was ineffective (*p* = 0.88) in preventing the degradation of anthocyanins. After 60 days of storage, no anthocyanins were found in either unprocessed or HHP-processed juices. Sinela et al. [29] investigated the degradation of anthocyanins of a hibiscus extract during storage, and reported that the degradation rate was much lower at temperatures of up to 20 °C (~20% degradation after 20 days). Since their extract was pasteurized, anthocyanins loss could only be attributed to non-enzymatic degradation. Therefore, in our juice, the high rate of anthocyanins loss during refrigerated storage is probably associated with enzymatic browning degradation, as studies in the literature show that this process still occurs at temperatures below 25 °C [30]. Although oxidative enzyme activity was not evaluated, we can hypothesize that HHP processing did not inactivate polyphenol oxidase, as reported by Brannan et al. [31] and González-Cebrino et al. [32] in papaya pulp and pumpkin purée, respectively. Jesus et al. [32] observed that inactivation of polyphenol oxidase only occurred when açaí pulp was HHP-processed at 65 °C. In that sense, HHP processing at temperatures higher than room temperature deserves to be investigated for jussara juice aiming at preserving anthocyanins.

The FRAP and TEAC values of jussara juices showed a gradual decrease during refrigerated storage, with 52% and 38% retention, respectively, by the end of 60 days (Figure 5, *p* < 0.0001). Nevertheless, FRAP and TEAC showed different trends during storage: the TEAC loss rate was constant during the whole storage period, whereas the FRAP loss rate decreased after 30 days of storage. This difference may be related to the significant association between TEAC values and anthocyanins content, as previously described. In that sense, the stability of FRAP values after 30 days of storage may be correlated to the presence of other phenolic compounds, possibly those formed as degradation products of anthocyanins (e.g., hydroxybenzoic acids derivatives).

While HHP processing did not affect FRAP loss during storage (*p* = 0.78), a negative effect was observed for TEAC values (*p* = 0.021). Even though this effect is of little relevance, it may be explained by a possible increase in polyphenol oxidase activity caused by HHP processing, as reported by Jesus et al. [33]. There are reports in the literature that HHP processing was ineffective at retaining the antioxidant activity of fruit smoothies stored at 4 °C for 30 days [34] and of aronia juice stored at 4 °C for 80 days [35].

It is important to note that microbiological analyses were not performed, being a limitation of this study.

## 4. Conclusions

HHP processing at 200 MPa for 5 min did not affect either the anthocyanins content or antioxidant activity (assessed by both FRAP and TEAC assays) of jussara juice. Sucrose addition at 12.5% was ideal for consumers’ acceptance and HHP processing did not impact the overall acceptance score. However, HHP processing was unable to preserve anthocyanins content and antioxidant activity during refrigerated storage for 60 days. Therefore, this study highlights the relevance of the production of ready-to-drink juices with jussara pulp, applying innovative processes such as HHP in order to maintain its functional composition and acceptance.

## Data Availability

The data presented in this study are available on request from the corresponding authors.

## Supplementary Materials

The following are available online at https://www.mdpi.com/article/10.3390/foods10102246/s1, Table S1: Experimental runs of the 2^2^ factorial design. Figure S1: Typical chromatographic separation of cyanidin-3-*O*-glucoside (peak 1) and cyanidin-3-*O*-rutinoside (peak 2) in jussara juice samples.
