# Supplementary material for: Effect of High Hydrostatic Pressure Processing on the Anthocyanins Content, Antioxidant Activity, Sensorial Acceptance and Stability of Jussara (Euterpe edulis) Juice
Source: Foods. 2021 Sep 22;10(10):2246. doi: 10.3390/foods10102246 (PMC8534504; doi:10.3390/foods10102246)
Supplement: Supplementary file 1 [file foods-10-02246-s001.zip › Supplementary Figure 1.pdf]

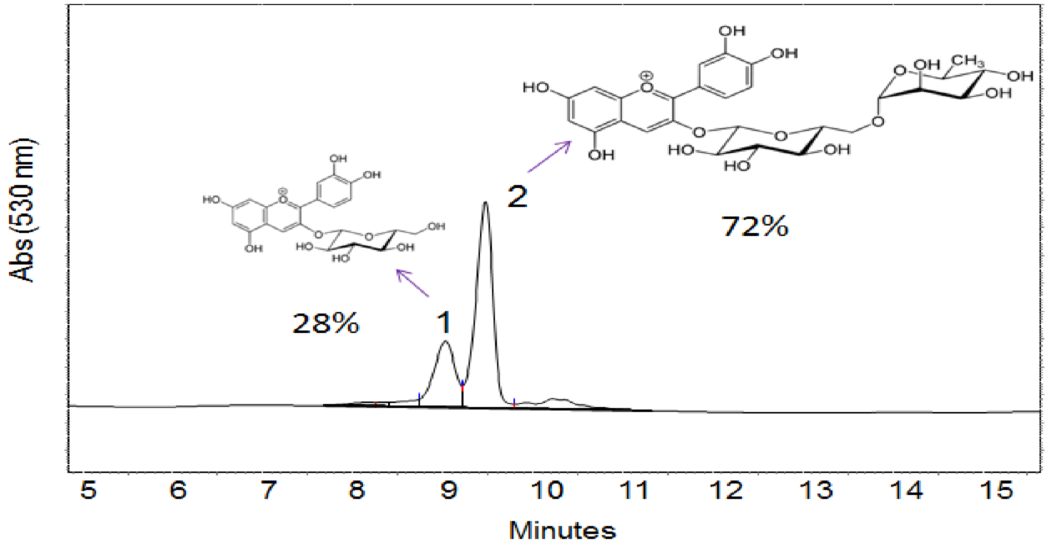

Figure S1. Typical chromatographic separation of cyanidin-3-*O*-glucoside (peak **1**) and cyanidin-3-*O*-rutinoside (peak **2**) in jussara juice samples.
