# Supplementary material for: Effect of High Hydrostatic Pressure Processing on the Anthocyanins Content, Antioxidant Activity, Sensorial Acceptance and Stability of Jussara (Euterpe edulis) Juice
Source: Foods. 2021 Sep 22;10(10):2246. doi: 10.3390/foods10102246 (PMC8534504; doi:10.3390/foods10102246)
Supplement: Supplementary file 1 [file foods-10-02246-s001.zip › Supplementary Table 1.pdf]

Table S1. Experimental runs of the 2^2 factorial design.

Run	Pressure (MPa)	Time (min)
1	200	5
2	500	5
3	200	10
4	500	10
5 (C)	350	7.5
6	200	5
7	500	5
8	200	10
9	500	10
10 (C)	350	7.5

(C) Central point
